# Association Between Gender and Payments Received From Pharmaceutical Companies Among Medical Professors and Associate Professors in Japan

**DOI:** 10.7759/cureus.58871

**Published:** 2024-04-23

**Authors:** Motoi Miura, Tetsuya Tanimoto, Hiroaki Saito, Kana Yamamoto, Erika Yamashita, Akihiko Ozaki

**Affiliations:** 1 Graduate School of Public Health, Teikyo University, Tokyo, JPN; 2 Internal Medicine, Navitas Clinic Kawasaki, Kawasaki, JPN; 3 Health Care, Medical Governance Research Institute, Tokyo, JPN; 4 Gastroenterology, Soma Central Hospital, Soma, JPN; 5 Medicine, Fukushima Medical University, Fukushima, JPN; 6 Breast and Thyroid Surgery, Jyoban Hospital of Tokiwa Foundation, Iwaki, JPN

**Keywords:** industry academia relationship, pharmaceutical companies, industry payment, ethics and professionalism, gender difference

## Abstract

Introduction

While prior research showed gender gaps in industry payments for medical professionals in the United States, there are limited data in Japan. So, this study seeks to investigate the potential gender gap in the receipt of pharmaceutical companies (PFCs) across all medical fields in Japan. Based on the results of previous studies, we developed a hypothesis that male doctors get more PFC than female doctors.

Materials and methods

Data from 92 pharmaceutical companies in Japan, covering 2016 to 2019, were analyzed. The analysis was conducted on professors and associate professors at all national and public medical universities in Japan, with gender as a factor variable and payments as an outcome variable, and variables that may have influenced the factor or outcome variables in previous studies, such as specialization, university type, region, rank and years since graduation, as control variables. Payments were converted to US dollars using the December 31, 2021, rate of 115 yen to the dollar for comparison purposes.

Results

Out of 1,825 subjects, 1,755 were males and 70 females. Males consistently received higher median payments from pharmaceutical companies (PFCs) than females across categories. In particular, among physicians specializing in internal medicine, the median PFC for men was $25 compared to $8 for women. For physicians affiliated with former imperial universities (the seven former imperial universities founded before World War II), the median PFC for men was $32 compared to $5 for women. Multivariate analysis confirmed significantly higher total benefits for males from 2016 to 2019, with the gender gap widening during this period (incidence rate ratio (IRR) for 2016: 0.51, IRR for 2019: 0.44).

Conclusions

Japanese male professors and associate professors received significantly higher PFCs than their female counterparts, and this gender gap expanded from 2016 to 2019, highlighting persistent gender inequality in the medical field in Japan, similar to trends observed in the United States.

## Introduction

Payments from industry play a significant role in medical research and clinical care [1-3]. For example, according to Boddapati et al., those receiving more than $100,000 per year from the industry had significantly more publications and a higher h-index for publication quality than those receiving less than $10,000 per year for all positions [2]. However, the receipt of money from industry, especially from pharmaceutical companies, also carries the risk of overprescribing expensive drugs [4-6]. Indeed, Inoue et al. investigated the association between industry payments to physicians and long-acting insulin prescribing behavior [4]. Specifically, the results showed that physicians who received industry payments prescribed $22,111 more long-acting insulin than those who did not [4].

Several factors that determine the amount of payment from pharmaceutical company (PFC) have been investigated, for example, job position, age, department of practice, etc. [7-9]. Also, gender is one of the factors. For instance, a previous study reported that among rheumatologists in the US, men received only $639 more in industry payments than women [7]. Moreover, a study examining pharmaceutical company funding among psychiatrists using the Centers for Medicare and Medicaid Services' Sunshine Act Open Payment database and the 2021 National Plan and Provider Enumeration System (NPPES) database found that women receive approximately 80% of the funding from pharmaceutical industry companies as men do [10].

However, a comprehensive survey covering all medical specialties has not yet been conducted on this topic in Japan. Japan has one of the lowest percentages of female physicians among the Organization for Economic Co-operation and Development (OECD) countries, so the difference in PFC is expected to be even larger due to the large gender gap [11]. Therefore, this study seeks to investigate the potential gender gap in the receipt of PFC across all medical fields in Japan. Based on the results of previous studies, we constructed the hypothesis that men obtain more PFC than women.

## Materials and methods

Study participants

We compiled payment data for lectures, writing and consulting from 92 pharmaceutical companies that disclosed payment data according to Japan Pharmaceutical Manufacturers Association (JPMA) guidelines for Japanese university medical schools from 2016 to 2019 [12]. Payment items such as meals, travel and lodging were excluded from the tabulation because they were disclosed in aggregate amounts. In this study, we focused specifically on professors and associate professors because prior research suggested that there may be disparities in the amount of PFC received by degree [13]. To complement this data, relevant subject information was collected from online sources and integrated into the database. Due to the unique regulations governing faculty members at private universities, this study included 42 national universities, eight public universities and the National Defense Medical College.

Dependent, independent and control variables

We employed gender as the independent variable and PFC as the dependent variable. We converted the PFC amounts to US dollars using an exchange rate of 115 yen to the dollar as of December 31, 2021. In addition, several variables that influence exposure factors or outcomes were employed as confounding factors with reference to previous study [14]. Specifically, we incorporated as control variables the department of specialty, the governing body of the affiliated university, the location of the affiliated university, job rank and the number of years since graduation as factors that may affect PFC in reference to previous studies [8,9,13,15,16].

Statistical analysis

We calculated summary statistics by gender for the variables collected as independent, dependent and control variables. We also calculated the median for each category of each variable by gender and compared whether there were differences between genders. In addition, to assess gender-based differences in PFC from 2016 to 2019, as well as for each individual year within that period, we applied a negative binomial distribution model to analyze the payments received by the individuals in our study. The significance level was set at 5% two-sided, and R statistical software (version 4.3.0, R Project for Statistical Computing, Vienna, Austria) was used for analysis [17].

Ethical approval

The Ethics Committee of the Medical Governance Research Institute approved the study (approval number: MG2018-04-20200605; approval date: June 5, 2020). Informed consent was waived due to this being a retrospective study that used publicly available data.

## Results

Study participants with one or more missing values in the items of the collected variables were excluded from the analysis, assuming that the missing values were randomly present. The final analysis included 1,825 subjects (1,755 males and 70 females). Table 1 shows the characteristics of the analyzed population by gender. Most of the population was male, and the median PFC was significantly higher for males; the specialty was mostly internal medicine for both males and females, and the operating organization of the institution where the population belonged to was mostly other public universities. Most of the population in the analysis were in non-professor positions, and most of the male and female population had graduated from 11 to 20 years.

**Table 1:** Characteristics of the analyzed population and median value for each attribute (1,000$) ^1^Median (interquartile range); n (%). ^2^Wilcoxon's rank sum test; Fisher's exact probability test; and Chi-square test.

Variables	Characteristics of the analyzed population			
	Total n = 1,825^1^	Male n = 1,755^1^	Female n = 70^1^	P value^2^
Amount (median (quartile range)) (1,000$)	21 (6, 73)	23 (6, 76)	8 (3, 28)	<0.001
Specialty	-	-	-	-
Internal Medicine	501 (27%)	490 (28%)	11 (16%)	-
Pediatrics	76 (4.2%)	67 (3.8%)	9 (13%)	-
Dermatology	58 (3.2%)	52 (3.0%)	6 (8.6%)	-
Psychiatry	44 (2.4%)	42 (2.4%)	2 (2.9%)	-
Surgery	380 (21%)	371 (21%)	9 (13%)	-
Orthopedics	85 (4.7%)	85 (4.8%)	0 (0%)	-
Obstetrics and Gynecology	74 (4.1%)	68 (3.9%)	6 (8.6%)	-
Ophthalmology	48 (2.6%)	45 (2.6%)	3 (4.3%)	-
Otorhinolaryngology	64 (3.5%)	64 (3.6%)	0 (0%)	-
Urology	48 (2.6%)	48 (2.7%)	0 (0%)	-
Neurosurgery	69 (3.8%)	69 (3.9%)	0 (0%)	-
Radiology	61 (3.3%)	59 (3.4%)	2 (2.9%)	-
Anesthesiology	42 (2.3%)	41 (2.3%)	1 (1.4%)	-
Pathology	115 (6.3%)	102 (5.8%)	13 (19%)	-
Laboratory Medicine	39 (2.1%)	34 (1.9%)	5 (7.1%)	-
Emergency Medicine	46 (2.5%)	46 (2.6%)	0 (0%)	-
Plastic Surgery	31 (1.7%)	31 (1.8%)	0 (0%)	-
Department of Rehabilitation	44 (2.4%)	41 (2.3%)	3 (4.3%)	-
Operating organization	-	-	-	0.4
Formerly an Imperial University	364 (20%)	347 (20%)	17 (24%)	-
Other public universities	1,461 (80%)	1,408 (80%)	53 (76%)	-
Region	-	-	-	0.4
Hokkaido・Tohoku	294 (16%)	283 (16%)	11 (16%)	-
Kanto・Chubu	585 (32%)	558 (32%)	27 (39%)	-
Kinki	423 (23%)	405 (23%)	18 (26%)	-
Chugoku・Shikoku	254 (14%)	245 (14%)	9 (13%)	-
Kyushu・Okinawa	269 (15%)	264 (15%)	5 (7.1%)	-
Position	-	-	-	<0.001
Professor	989 (54%)	968 (55%)	21 (30%)	-
Others	836 (46%)	787 (45%)	49 (70%)	-
Years since graduation	-	-	-	<0.001
10 or less than 10 years	22 (1.2%)	17 (1.0%)	5 (7.1%)	-
11-20 years	1,603 (88%)	1,552 (88%)	51 (73%)	-
More than 20 years	200 (11%)	186 (11%)	14 (20%)	-

Table 2 shows the median for each attribute by gender. The median was significantly higher for men in most attributes, especially for those from former imperial universities (median (Q1, Q3): $32,000 ($9,000, $93,000) vs. $5,000 ($1,000, $29,000)). When looking at the medians calculated for each variable’s category, significant differences between men and women existed in most categories, but there were no significant differences between men and women in the median PFC for those specializing in surgery; those belonging to universities in the Kinki, Kyushu and Okinawa regions; and those in the position of professor.

**Table 2:** Median for each attribute by gender ($1,000) ^1^Median (interquartile range); n (%). ^2^Wilcoxon's rank sum test; Fisher's exact probability test; and Chi-square test. *Internal Medicine, Pediatrics, Dermatology, Psychiatry, Obstetrics and Gynecology Ophthalmology, Otorhinolaryngology, Urology, Radiology, Anesthesiology, Pathology, Laboratory Medicine, Emergency Medicine and Department of Rehabilitation. **Surgery, Orthopedics, Neurosurgery and Plastic Surgery.

Variables	Male n = 1,755^1^	Female n = 70^1^	P value^2^
Specialty	-	-	-
Internal Medicine*	25 (6, 87)	8 (3, 20)	<0.01
Surgical Specialties**	20 (6, 53)	13 (4, 92)	0.64
Operating organization	-	-	-
Formerly an Imperial University	32 (9, 93)	5 (1, 29)	<0.01
Other public universities	21 (6, 68)	8 (3, 24)	<0.01
Region	-	-	-
Hokkaido・Tohoku	24 (8, 72)	5 (1, 16)	0.014
Kanto・Chubu	21 (5, 77)	6 (1, 29)	<0.01
Kinki	23 (6, 78)	9 (4, 42)	0.09
Chugoku・Shikoku	25 (5, 78)	5 (3, 9)	0.01
Kyushu・Okinawa	23 (6, 68)	14 (4, 24)	0.32
Position	-	-	-
Professor	40 (11, 111)	43 (5, 78)	0.16
Others	11 (4, 37)	4 (1, 14)	<0.01
Years since graduation	-	-	-
10 or less than 10 years	10 (6, 39)	1 (1, 1)	0.02
11-20 years	24 (6, 81)	8 (4, 35)	<0.01
More than 20 years	14 (3, 52)	3 (1, 9)	<0.01

The results of the multivariate analysis are presented in Table 3. The multivariate analysis reveals that even after adjusting for potential confounding factors, the total amount received was still significantly higher for men from 2016 to 2019 (incidence rate ratio (IRR): 0.41, 95% confidence interval (95% CI): 0.30, 0.56). Further analysis for each year from 2016 to 2019 suggested that the IRR expanded from 2016 to 2019 (IRR in 2016: 0.51, IRR in 2019: 0.44). This suggests that the gender difference in PFC receipt may have widened from 2016 to 2019.

**Table 3:** Association between gender and the amount of money received from pharmaceutical companies analyzed using the negative binominal model and the disparity in the amount of money from 2016 to 2019 *Incidence rate ratio. **Confidence interval. ***Internal Medicine, Pediatrics, Dermatology, Psychiatry, Obstetrics and Gynecology Ophthalmology, Otorhinolaryngology, Urology, Radiology, Anesthesiology, Pathology, Laboratory Medicine and Emergency Medicine. ****Surgery, Orthopedic Surgery, Neurosurgery and Plastic Surgery.

Variables	2016-2019			2016			2017			2018			2019		
	IRR*	95% CI**	P value	IRR*	95% CI**	P value	IRR*	95% CI**	P value	IRR*	95% CI**	P value	IRR*	95% CI**	P value
Gender	-	-	-	-	-	-	-	-	-	-	-	-	-	-	-
Male	Ref	-	-	Ref	-	-	Ref	-	-	Ref	-	-	Ref	-	-
Female	0.41	0.3, 0.56	<0.001	0.51	0.38, 0.7	<0.001	0.48	0.33, 0.66	<0.001	0.45	0.33, 0.64	<0.001	0.44	0.33, 0.61	<0.001
Region	-	-	-	-	-	-	-	-	-	-	-	-	-	-	-
Hokkaido・Tohoku	Ref	-	-	Ref	-	-	Ref	-	-	Ref	-	-	Ref	-	-
Kanto・Chubu	1.09	0.92, 1.3	0.3	1.12	0.94, 1.31	0.2	1.11	0.93, 1.31	0.2	1.16	0.98, 1.38	0.089	1.13	0.94, 1.34	0.2
Kinki	1.11	0.91, 1.32	0.3	1.11	0.93, 1.31	0.2	1.12	0.93, 1.34	0.2	1.11	0.92, 1.34	0.3	1.13	0.93, 1.35	0.2
Chugoku・Shikoku	1.09	0.88, 1.35	0.4	1.06	0.87, 1.3	0.5	1.09	0.9, 1.35	0.4	1.15	0.93, 1.42	0.2	1.2	0.97, 1.49	0.093
Kyushu・Okinawa	1.04	0.85, 1.28	0.7	1.08	0.9, 1.31	0.4	0.98	0.8, 1.2	0.8	1.08	0.88, 1.32	0.5	1.11	0.9, 1.35	0.4
Specialty	-	-	-	-	-	-	-	-	-	-	-	-	-	-	-
Internal Medicine	Ref	-	-	Ref	-	-	Ref	-	-	Ref	-	-	Ref	-	-
Surgical Specialties	0.68	0.6, 0.77	<0.001	0.64	0.57, 0.71	<0.001	0.67	0.59, 0.76	<0.001	0.67	0.59, 0.76	<0.001	0.67	0.59, 0.76	<0.001
Position	-	-	-	-	-	-	-	-	-	-	-	-	-	-	-
Professor	Ref	-	-	Ref	-	-	Ref	-	-	Ref	-	-	Ref	-	-
Others	0.39	0.33, 0.44	<0.001	0.33	0.3, 0.37	<0.001	0.38	0.33, 0.42	<0.001	0.44	0.39, 0.49	<0.001	0.5	0.44, 0.56	<0.001
Operating organization	-	-	-	-	-	-	-	-	-	-	-	-	-	-	-
Formerly an Imperial University	Ref	-	-	Ref	-	-	Ref	-	-	Ref	-	-	Ref	-	-
Other public universities	0.71	0.61, 0.82	<0.001	0.77	0.68, 0.89	<0.001	0.76	0.66, 0.88	<0.001	0.68	0.59, 0.79	<0.001	0.68	0.59, 0.79	<0.001
Years since graduation	-	-	-	-	-	-	-	-	-	-	-	-	-	-	-
10 or less than 10 years	Ref	-	-	Ref	-	-	Ref	-	-	Ref	-	-	Ref	-	-
11-20 years	1.25	0.7, 2.03	0.4	1.7	0.96, 2.72	0.051	1.38	0.76, 2.34	0.2	1.31	0.72, 2.23	0.3	0.84	0.46, 1.42	0.5
More than 20 years	1.06	0.59, 1.77	0.8	1.48	0.83, 2.53	0.2	1.15	0.63, 1.99	0.6	1.28	0.69, 2.23	0.4	0.79	0.42, 1.36	0.4

## Discussion

Summary of results

When the PFC for 2016-2019 was pooled and analyzed, male Japanese professors and associate professors received more PFC than their female counterparts. Moreover, it was also noted that the difference in amounts between genders may have increased more between 2016 and 2019.

Concordance and differences with previous studies

The amount of money received from industry was similar to the results of previous studies, which showed that women received less than men [7,10,18,19]. However, the results differed from those of previous studies in several respects. For example, compared to previous studies, the differences between men and women in the amount of money received from firms were relatively large. We expect that the reasons for these results are as follows. In Japan, professional relationships with pharmaceutical companies often develop outside regular working hours [20]. Given societal expectations that primarily assign women to childcare and domestic responsibilities, even female medical professionals usually have limited availability outside of regular working hours [21]. This leads to fewer opportunities for women to establish industry connections, which subsequently results in fewer payments from pharmaceutical companies. Also, unlike previous studies, the region in which the facilities belonging to the study participants were located had no significant effect on PFC. We speculate that this is largely due to the fact that medical schools are not unevenly distributed in Japan because of the government's policy of establishing approximately one medical school per prefecture [22].

Clinical implications

From our results, several clinical implications can be obtained. The first is that since women received significantly lower amounts of PFC, it is possible that women are better able to provide quality care because they are less tied to pharmaceutical companies. In fact, several studies have shown that there are differences in outcomes and quality of treatment between men and women [23,24]. For example, Tsugawa et al. compared mortality and readmission rates for approximately 150,000 general internal medicine patients treated by Medicare with those treated by female and male physicians [23]. The analysis showed that older hospitalized patients treated by female internists had lower mortality and readmission rates than those treated by male internists.

Additionally, we noted an increasing disparity in the distribution of payments between men and women during the study period. This result implies the need for policies in Japan to promote gender equality in various aspects of healthcare. Despite global advancements in gender equity, Japan has seen limited progress in narrowing its gender gap index [25]. Considering this, the observed gender disparity within the traditionally male-dominated Japanese medical field is not surprising. Given the global challenges faced by women in academia and the importance of gender equity in scientific advancement during the COVID-19 pandemic, this disparity in Japan could have been amplified in recent times [26,27].

Limitation

The study has several limitations. First, the findings may have limited external validity due to the study's focus on national and public universities in Japan. Second, manual recording of payments to healthcare providers may have resulted in classification errors. In the future, when PFC reporting becomes mandatory and it is compiled into a database, as it is in the US, a more accurate analysis could be conducted [28]. The third limitation is the possibility of unmeasured confounding factors. If we add them to the analysis in the future, the strength of the association and other factors may change.

## Conclusions

The study investigated the gender gap in pharmaceutical company payments to professors and associate professors in Japan from 2016 to 2019. Results indicated a significant disparity, with male recipients receiving substantially higher amounts of PFC compared to female recipients. Furthermore, the gap appeared to widen over the study period, suggesting a concerning trend of increasing inequality. The findings underscore the importance of addressing gender disparities in pharmaceutical industry payments within the medical field. Female doctors receive significantly less PFC, which may reduce their influence from pharmaceutical companies with regard to prescribing practices. This may affect the quality of care they provide. Additionally, the widening gap highlights the urgent need for policy interventions to promote gender equality in healthcare in Japan, particularly in light of global efforts toward gender equity and the challenges exacerbated by the COVID-19 pandemic.
